# Endogenous and Recombinant Type I Interferons and Disease Activity in Multiple Sclerosis

**DOI:** 10.1371/journal.pone.0035927

**Published:** 2012-06-06

**Authors:** Finn Sellebjerg, Martin Krakauer, Signe Limborg, Dan Hesse, Henrik Lund, Annika Langkilde, Helle Bach Søndergaard, Per Soelberg Sørensen

**Affiliations:** 1 Danish Multiple Sclerosis Center, Department of Neurology, Copenhagen University Hospital Rigshospitalet, Copenhagen, Denmark; 2 Danish Research Centre for Magnetic Resonance, Section 340, Copenhagen University Hospital Hvidovre, Hvidovre, Denmark; 3 Department of Radiology, Copenhagen University Hospital Rigshopitalet, Copenhagen, Denmark; University Hospital La Paz, Spain

## Abstract

Although treatment of multiple sclerosis (MS) with the type I interferon (IFN) IFN-β lowers disease activity, the role of endogenous type I IFN in MS remains controversial. We studied CD4+ T cells and CD4+ T cell subsets, monocytes and dendritic cells by flow cytometry and analysed the relationship with endogenous type I IFN-like activity, the effect of IFN-β therapy, and clinical and magnetic resonance imaging (MRI) disease activity in MS patients. Endogenous type I IFN activity was associated with decreased expression of the integrin subunit CD49d (VLA-4) on CD4+CD26^high^ T cells (Th1 helper cells), and this effect was associated with less MRI disease activity. IFN-β therapy reduced CD49d expression on CD4+CD26^high^ T cells, and the percentage of CD4+CD26^high^ T cells that were CD49d^high^ correlated with clinical and MRI disease activity in patients treated with IFN-β. Treatment with IFN-β also increased the percentage of CD4+ T cells expressing CD71 and HLA-DR (activated T cells), and this was associated with an increased risk of clinical disease activity. In contrast, induction of CD71 and HLA-DR was not observed in untreated MS patients with evidence of endogenous type IFN I activity. In conclusion, the effects of IFN-β treatment and endogenous type I IFN activity on VLA-4 expression are similar and associated with control of disease activity. However, immune-activating effects of treatment with IFN-β may counteract the beneficial effects of treatment and cause an insufficient response to therapy.

## Introduction

The type I interferons (IFNs) IFN-α and IFN-β are produced in response to viral infections and induce changes in cellular function by binding to specific receptors on the cell surface, resulting in the induction or repression of numerous genes and a wide range of antiviral and immunological effects [1], [2]. Treatment with recombinant interferon IFN-β decreases disease activity in relapsing-remitting multiple sclerosis (MS) by approximately 30% [3]–[5].

Recent studies have identified an endogenous type I IFN gene expression signature in a subgroup of untreated patients with MS [6], [7]. This response has been linked with expression of the immunoregulatory cytokine interleukin (IL)-10, the immunoregulatory transcription factor FoxP3 and protection from disease activity in untreated MS patients and during subsequent treatment with IFN-β [8]–[10]. Furthermore, the expression of IL-10 is lower in patients who have developed neutralizing antibodies to IFN-β than in other untreated MS patients and healthy control subjects, suggesting that endogenous IFN-β is involved in the induction of IL-10 [9]. Other studies have, however, suggested that the expression of IFN-stimulated genes in untreated MS patients is associated with a diminished capacity to induce IFN-stimulated genes and a higher risk of breakthrough disease upon subsequent treatment with exogenous IFN-β [11]. The reason for these differences, which indicate completely different roles of endogenous and exogenous IFN-β in the pathogenesis of MS, is unknown.

To further explore this subject, we compared the effect of IFN-β treatment with the effects associated with evidence of endogenous type I IFN activity on CD4+ T cell and T cell subset activation, monocyte and DC activation and clinical and magnetic resonance imaging disease activity in MS. The CD4+ T cell subsets studied were identified according to their expression of CD25 (the IL-2 receptor α-chain) and CD26. CD25 is expressed at high levels on regulatory T cells but also on highly activated effector T cells [12], [13]. CD26 expression identifies a subset of CD4+ T cells with a T helper type 1 (Th1) phenotype previously implicated in the pathogenesis of MS [14]–[17]. This study identifies parallels between the effects of endogenous type I IFN-like activity and the effects of treatment with IFN-β that are associated with a reduction in disease activity. However, IFN-β therapy is also found to have additional effects on circulating T cells, some of which are associated with an unexpected increase in the risk of disease activity.

## Materials and Methods

### Patient Material

The study was approved by the regional ethics committee of Copenhagen and Frederiksberg (KF01–041/95). All patients provided written informed consent. Venous blood samples were obtained from 39 untreated patients with relapsing-remitting MS of whom 24 subsequently began treatment with IFN-β (IFN-β1a 30 µg once weekly in 19 patients, IFN-β1a 44 µg three times weekly in 4 patients and IFN-β1b 250 µg every other day in one patient). These patients were followed with serial blood samples after three and six months of therapy [8]. One patient who developed neutralizing anti-IFN-β antibodies after six months of therapy was excluded from the subsequent analysis. Blood samples from this cohort were obtained 9–12 hours after an injection of IFN-β. In addition, we studied 40 patients who had been treated with IFN-β for at least six months (including 14 patients who were also included in the first cohort); 20 of these patients were treated with IFN-β1a 30 µg once weekly and 20 received IFN-β1a 44 µg three times weekly. Blood samples from these patients were obtained 36–48 hours after an injection of IFN-β. Further description of the cohorts is presented in Table 1. A control group consisting of 12 healthy volunteers (8 women and 4 men; median age 31 years, range 26–46 years) was also included in the study.

**Table 1:** Overview of the patient material.

	Untreated (n = 39)	Interferon-treated (early cohort, n = 23)	Interferon-treated (late cohort, n = 40)
Median age (range)	33 years (23–53)	30 years (23–46)	33 years (23–57)
Gender	24 women/15 men	15 women/8 men	26 women/14 men
Median duration of disease	4 years (1–25)	2 years (1–12)	5.5 years (1–20)
Median Kurtzke EDSS score (range)	2.0 (0–6.0)	1.0 (0–6.0)	2.0 (0–6.0)
Duration of treatment	–	–	2 years (0.5–9.5)
Median relapse rate year prior to study inclusion (range)	1/year (0–4)	1/year (0–4)	0/year (0–4)

### Assessment of Disease Activity

Clinical activity was defined as the occurrence of a confirmed relapse (new or recurrence of previous symptoms of MS lasting >24 hours, in the absence of fever or signs of systemic infection, and with findings on neurological examination consistent with the symptoms). All patients were followed up with biannual control visits for one year and acute visits in case of new symptoms suggesting a relapse. Time to first relapse was established for all patients.

### Magnetic Resonance Imaging

MRI was performed using a 3.0 T whole body scanner (Trio, Siemens, Erlangen, Germany), maximum gradients 40 mT/m, 8-channel head coil within one week of all blood samplings [8]. To quantify the lesion load, fluid-attenuated inversion recovery (FLAIR) and proton density/T2 weighted 2D imaging sequences were used. A T1-weighted 3D imaging sequence (Magnetization Prepared RApid Gradient Echo (MPRAGE) was acquired approximately 15 minutes post-intravenous gadolinium (Gd) administration (0.2 mmol/kg body weight of Magnevist (Schering AG, Berlin, Germany)). T2 lesions and enhancing lesions were detected and counted by an experienced technician using in-house developed software.

### Flow Cytometry Analysis of CD4+ T Cells and Antigen-presenting Cells

A lyse-and-wash whole blood staining procedure, where whole blood samples were stained with a cocktail of pretitrated fluoresceinated monoclonal antibodies, lysed, washed and analyzed on a BD FACSCalibur™ flow cytometer, was used to analyse CD4+, CD25^high^ and CD26^high^ T cells and CD14+ monocytes. Staining with anti-HLA-DR, a lineage antibody cocktail, anti-CD11a (expressed on myeloid dendritic cells) and anti-CD123 (expressed on plasmacytoid dendritic cells) antibodies were used for the identification of dendritic cells. Isotype control reagents and unstained controls were used to control for non-specific antibody binding and autofluorescence. Analysis of list-mode files was conducted with the BD FACSDiva™ software (Becton Dickinson). A list of all surface markers studied is given in Table S1.

### In vitro Analysis of T Cell Activation

Blood mononuclear cells (MNCs) were isolated from heparinised venous blood from 11 healthy volunteers using density gradient centrifugation of heparinised venous blood on Lymphoprep. All incubations were performed at 37°C with 5% CO_2_ in RPMI1640 medium (Invitrogen, Taastrup, Denmark) supplemented with 5% human serum albumin. The cells were cultured for 24 hours in 6-well plates at a concentration of 2×10^6^ cells/ml with 1 ng/ml recombinant human IFN-β1a (Avonex, Biogen-Idec, Cambridge, MA, USA), 10 ng/ml 6-α-methylprednisolone (MP) dissolved in dimethylsulfoxide (Sigma), IFN-β1a and MP, or none of these drugs. After 24 hours the cells were washed, and CD4+ and CD8+ T cells’ expression of CD25, CD69, CD71 and annexin V binding (a measure of apoptotic cell death) was measured by flow cytometry and *FOXP3* gene expression was measured by PCR analysis.

### Gene Expression Analysis

Expression of *MX1* (encoding the type I interferon-induced myxovirus resistance A molecule), *FOXP3* and the chemokine *CXCL10* was measured in whole blood cells or cultured MNCs by quantitative real-time polymerase chain reaction (PCR) analysis as previously described [9], [18].

### Statistical Analysis

The sample size was based on previous studies on the effects of IFN-β in small patient cohorts and on studies reporting that gene expression profiling in 22 MS patients could identify subgroups with different disease courses during treatment with IFN-β [19]–[21]. Values are given as median with inter-quartile range. Statistical testing was by the Wilcoxon test, the Mann-Whitney U-test, paired t-test, non-parametric correlation analysis (Spearman rank correlation coefficient [SRCC]), Cox regression analysis (hazard ratio with 95% confidence interval), the log-rank test and Kaplan-Meier plots. P<0.05 (two-sided testing) was considered significant; a Bonferroni correction was applied to post-hoc Man-Whitney tests after Wilcoxon analyses.

## Results

### Circulating APCs and CD4+ T Cells

Anti-CD25 and anti-CD26 antibody staining identified a population of CD25^high^ CD4+ T cells with intermediate expression of CD26 and a population of CD26^high^ CD4+ T cells with intermediate expression of CD25 (Figure S1). The CD4+CD25^high^ population constituted 2.1% (IQR 0.5%) of CD4+ T cells and the CD4+CD26^high^ population constituted 12% (IQR 6.8%) of the CD4+ T cells in blood from healthy controls. There was no difference in the percentage of these subsets between MS patients and controls, but MS patients had a higher percentage of CD4+CD25^high^ T cells expressing CCR5 (Figure S2). MS patients also had a higher percentage of DCs expressing CD80 than did healthy controls (Figure S2). There was no difference in the absolute number of circulating leukocytes, lymphocytes, monocytes, dendritic cells, CD3+ T cells or CD4+ T cells in untreated MS patients and healthy controls, and the expression of all other molecules studied was comparable in untreated MS patients and healthy controls (data not shown).

### Endogenous Type I IFN Activity in Untreated MS

**Figure 1:**
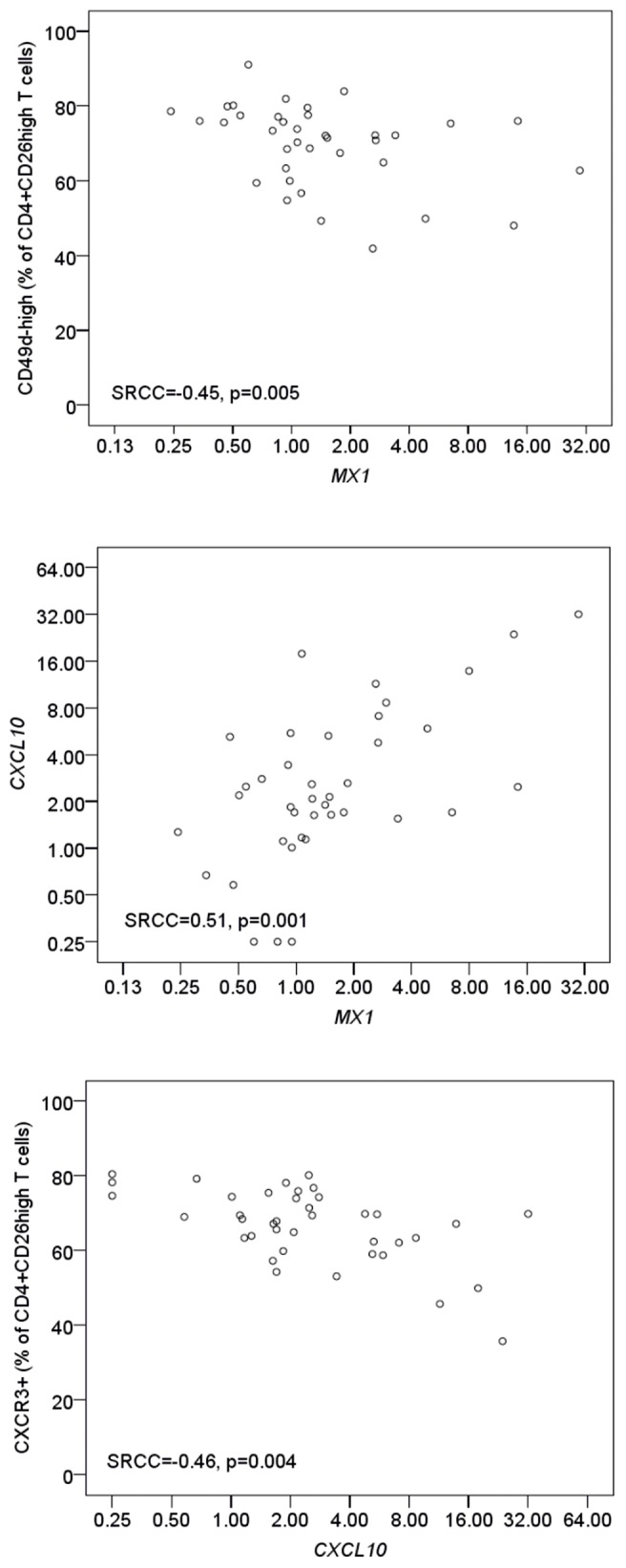
T cell activation, *CXCL10* and *MX1* expression. The relationship between the percentage of CD4+CD26^high^ T cells expressing CD49d or CXCR3 and the expression of *MX1* and *CXCL10* mRNA in blood mononuclear cells from untreated patients with relapsing-remitting multiple sclerosis was analysed by Spearman rank correlation coefficients (SRCC).

The expression of *MX1* mRNA was associated with a type I IFN-induced gene expression signature in 36 of the untreated MS patients included in the present study [9]. The percentage of CD4+CD26^high^ T cells that were CD49d^high^ correlated negatively with expression of *MX1* mRNA in blood cells (Figure 1). Expression of *FOXP3* in blood cells also correlated negatively with the percentage of CD49d^high^ CD4+ T cells (SRCC = −0.50, p = 0.002), whereas there was no correlation with *IL10* expression (data not shown). There were no other significant relationships between *MX1* gene expression and CD4+ T cell or APC activation, but the expression of *MX1* correlated with expression of the chemokine *CXCL10* (Figure 1) which, in turn, correlated negatively with the percentage of CD4+CD26^high^ T cells that expressed the CXCL10 receptor CXCR3 (Figure 1).

### Effects of IFN-β on CD4+ T Cell and APC Activation

Treatment with IFN-β transiently decreased the number of circulating lymphocytes, dendritic cells and CD3+ and CD4+ T cells and increased the number of monocytes 9–12 hours after an injection of IFN-β (Table S2). These effects were not observed 36–48 hours post-injection. Other effects of IFN-β were persistent as they were observed both 9–12 and 36–48 hours after an injection of IFN-β: an increased percentage of CD4+ and CD4+CD25^high^ T cells expressing CD71; an increased percentage of CD4+ T cells expressing HLA-DR; a decreased percentage of CD4+CD26^high^ T cells that were CXCR3+ or CD49d^high^; an increased percentage of monocytes expressing CD86; and a decreased percentage of dendritic cells expressing CCR5 (Table S2). Other statistically significant effects observed either 9–12 or 36–48 hours post-injection are summarized in Table S2. There were no significant differences between the patients treated with IFN-β1a 30 µg once weekly and patients treated with IFN-β1a 44 µg three times weekly.

### IFN-β Treatment and MRI Disease Activity

At the six months scan 7/23 patients (30%) had one or more Gd-enhancing lesions on MRI (median 1, range 1–12 lesions) and 15/23 patients had one or more new or enlarged T2-weighted MRI lesions during the six months of follow-up (median 3, range 1–27 lesions). The relationship between the number of Gd-enhancing lesions at month 6 and the number of new or enlarged T2 lesions and the immunological variables is shown in Table 2. The percentage of CD4+CD26^high^ T cells that were CD49d^high^ correlated positively with the number of Gd-enhancing MRI lesions (SRCC = 0.58, p = 0.006) and the number of new or enlarged T2 lesions (SRCC = 0.46, p = 0.042). The percentage of CD4+CD25^high^ T cells that were CD137+ correlated negatively with the number of Gd-enhancing lesions (SRCC = −0.47, p = 0.025) and the number of new or enlarged T2 lesions (SRCC = −0.64, p = 0.001).

**Table 2:** Immune activation and disease activity.

	Hazard ratio of relapse	Gd-enhancing lesions	New or enlarged T2 lesions
Dendritic cells			
CD40 positive (%)	1.39 (1.12–1.73), p = 0.003	0.31, NS	0.42, NS
CD80 positive (%)	1.10 (1.01–1.20), p = 0.033	0.30, NS	0.11, NS
CD4+ T cells			
CD62L^high^	0.83 (0.69–1.00), p = 0.049	−0.24, NS	− `0.51, p = 0.023
CD71+	1.38 (1.10–1.73), p = 0.005	0.11, NS	0.05, NS
CD95+	1.18 (1.04–1.34), p = 0.009	0.10, NS	0.03, NS
HLA-DR+	1.32 (1.06–1.63), p = 0.014	0.00, NS	0.16, NS
CD4+CD25^high^ T cells			
CD137+	0.68 (0.51–0.91), p = 0.009	−0.47, p = 0.025	−0.64, p = 0.001
CD4+CD26^high^ T cells			
CD49d^high^	1.21 (1.01–1.43), p = 0.029	0.58, p = 0.006	0.46, p = 0.042

Relapse risk, magnetic resonance imaging disease activity, T cell and dendritic cell activation in blood samples obtained 9–12 hours after an injection of interferon-β in 23 MS patients treated with interferon-β for six months.

### IFN-β Treatment and Clinical Disease Activity

In univariate Cox regression analyses the one-year relapse risk was associated with several variables in samples obtained early after an injection of IFN-β after 6 months of treatment: the percentage of dendritic cells expressing CD40 and CD80; the percentage of CD4+ T cells that were CD62L^high^ or expressed CD71, CD95 or HLA-DR; the percentage of CD4+CD25^high^ cells that expressed CD137; and the percentage of CD4+CD26^high^ cells that were CD49d^high^ (Table 2). In multivariate analyses the percentage of CD4+ T cells expressing HLA-DR (hazard ratio 1.89 [1.13–3.16], p = 0.015) and the percentage of dendritic cells expressing CD40 (hazard ratio 1.84 [1.16–2.90], p = 0.009) were independent predictors of relapse risk. Although MRI disease activity was associated with relapse risk in a univariate analysis [8], Gd-enhancing MRI lesions were not an independent predictor of relapse risk. Neither did baseline flow cytometry results correlate with disease activity on subsequent treatment with IFN-β (data not shown).

**Figure 2:**
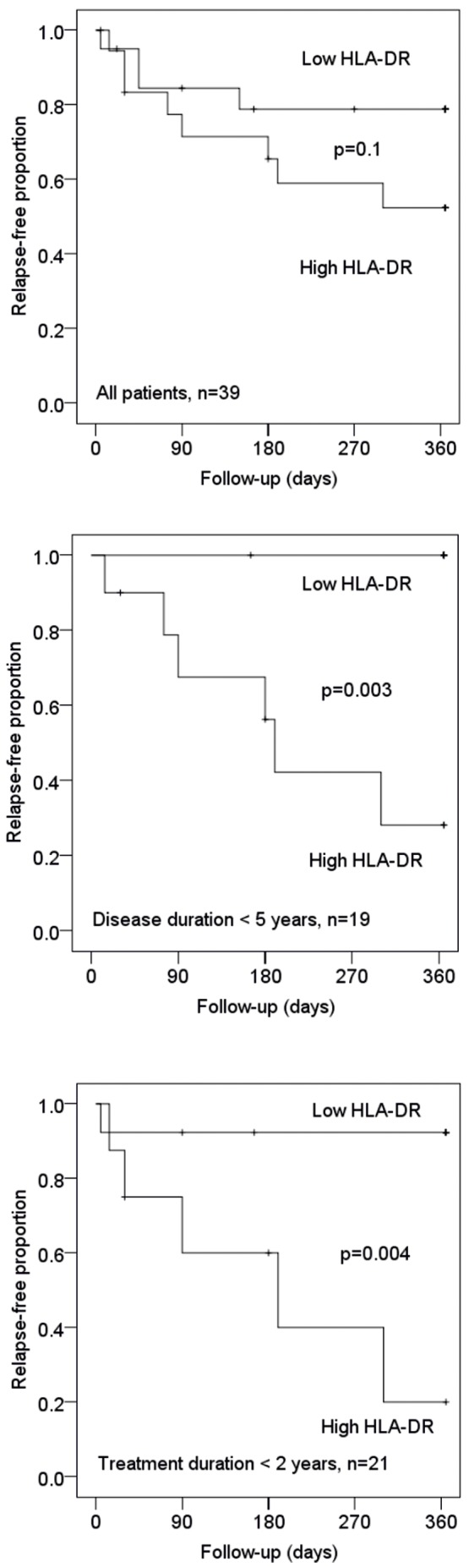
T cell activation and relapse risk. Relationship between CD4+ T cell expression of HLA-DR and relapse risk in 39 patients from whom blood samples were obtained 36–48 hours after an injection of interferon-β. Patients were dichotomized around the median and relapse risk was analysed in Kaplan-Meier plots and with the log-rank test in all patients and in subgroups of patients with a shorter duration of treatment or disease duration.

The relationship between CD40 expression on dendritic cells and HLA-DR expression on CD4+ T cells and relapse risk was further analysed in the 40 patients from whom blood samples were obtained 36–48 hours after an injection of IFN-β. In this cohort there was no relationship between CD40 expression on dendritic cells and relapse risk. However, patients with a percentage of HLA-DR positive CD4+ T cells above median had a higher relapse risk than patients with below median values (Figure 2). This relationship was highly significant in patients with a disease duration of less than 5 years (n = 19, p = 0.003) and in patients treated with IFN-β for less than two years (n = 21, p = 0.004) (Figure 2). Gd-enhancing MRI lesions were observed in 10/40 of these patients but were not an independent predictor of relapse risk in the multivariate analysis.

### Correlation between Flow Cytometry Measurements

Comparing values obtained 9–12 hours after an injection of IFN-β after six months of treatment we found moderate positive correlations (SRCC>0.5) between the percentage of CD4+ T cells expressing CD95 and HLA-DR (SRCC = 0.71, p<0.001) and CD71 (SRCC = 0.73, p<0.001); between the percentage of CD4+CD25^high^ T cells expressing CD137 and the percentage of CD4+ T cells that were CD62L^high^; and a negative correlation between the percentage of CD4+CD25^high^ T cells expressing CD137 and the percentage of CD4+CD26high T cells that were CD49d^high^.

The variability of the flow cytometry measurements was analysed by comparing the results of the flow cytometry measurements after three and six months of therapy. The percentage of CD4+ T cells expressing HLA-DR (SRCC = 0.71, p<0.001), CD95 (SRCC = 0.51, p = 0.027) or that were CD62L^high^ (SRCC = 0.63, p = 0.003) correlated after three and six months of therapy (data not shown). There were no significant correlations between month 3 and month 6 results for the other variables studied.

### In vitro Studies of T Cell Activation by Interferon-β

The effect of IFN-β and MP on CD4+ T cells was studied after 24 hours of *in vitro* treatment of MNCs from healthy volunteers (Table 3). Treatment with IFN-β or IFN-β in combination with MP did not affect the viability of the cells as assessed by annexin V staining, but MP increased the percentage of annexin V-binding CD4+ T cells (p<0.001). Incubation with IFN-β increased the percentage of CD4+ T cells that were CD25+ (p<0.001), CD25^high^ (p<0.001) and CD71+ (p = 0.01) and the expression of *FOXP3* mRNA in MNCs (p = 0.005) compared with untreated cells. Treatment with MP alone reduced the percentage of CD4+ T cells that were CD25^high^ (p = 0.002) or CD71+ (p = 0.011) and reduced *FOXP3* expression (p = 0.024) compared with untreated cells. Combination treatment with IFN-β and MP increased the percentage of CD4+ that cells that were CD25+ T cells but had no effect on CD25^high^ or CD71+ CD4+ T cells or *FOXP3* mRNA expression.

**Table 3:** Ex vivo effect of interferon-β1a and methylprednisolone.

	Control	IFN-β	MP	IFN-β and MP
CD4+Annexin V+	5.6% (1.4)	5.0% (2.3), NS	8.9% (1.5), p<0.001	6.0% (1.7), NS
CD4+CD25+	21% (2.3)	26% (2.3), p<0.001	20% (1.9), NS	24% (2.7), p = 0.007
CD4+CD25^high^	2.8% (0.23)	3.7% (0.39), p<0.001	1.9% (0.22), p = 0.002	3.1 (0.26), NS
CD4+CD71+	0.54% (0.11)	0.77% (0.22), p = 0.01	0.40% (0.08), p = 0.011	0.44% (0.07), NS
*FOXP3* mRNA	2.8 (0.7)	6.2 (1.2), p = 0.005	0.93 (0.86), p = 0.024	2.0 (1.3), NS

Effect of *ex vivo* treatment of blood mononuclear cells (MNCs, n = 11) with interferon-β1a (IFN-β) and/or methylprednisolone (MP) for 24 hours on surface expression of CD25 and CD71 on CD4+ T cells and expression of *FOXP3* mRNA.

## Discussion

The major findings in the present study are that: 1) endogenous type I IFN-like activity and treatment with IFN-β are both associated with reduced expression of CD49d on CD26^high^ CD4+ T cells (Th1 helper cells) and this correlates with MRI disease activity in IFN-β-treated MS patients; 2) treatment with IFN-β also induces activation of CD4+ T cells, as evidenced by the induction of CD71 and HLA-DR, and this is associated with an increased relapse risk.

CD4+CD26^high^ T cells are enriched for expression of Th1 markers and produce high levels of tumour necrosis factor (TNF)-α and IFN-γ [14], [15]. Disease activity in relapsing-remitting MS and in patients with clinically isolated syndromes is associated with an increased percentage of circulating CD4+CD26+ T cells [14], [16], [17]. These findings indicate that CD26^high^ T cells are a potential target for immunomodulatory MS treatments, and in a previous study we found that the number of circulating T cells, especially CD4+CD26+ T cells, predicted relapses in MS patients treated with IFN-β [22]. CD49d is an integrin α-chain that together with the integrin β-chain CD29 molecule forms very late antigen (VLA)-4. The role of VLA-4 in the pathogenesis of MS is clearly evidenced by the efficacy of treatment with the anti-CD49d molecule natalizumab. CD49d is expressed at high levels on CD4+CD26^high^ T cells, and CD4+CD49d^high^ T cells are increased in patients with active MS [15], [23]. IFN-β decreases the expression of CD49d in MS [20], [24]. We extend these findings by showing that a decrease in CD49d^high^ cells is found mainly in CD4+CD26^high^ cells in patients treated with IFN-β and that endogenous type I IFN activity, as assessed by expression of *MX1*, correlates negatively with the percentage of CD4+CD26^high^ T cells that are CD49d^high^. These findings suggest that not only IFN-β treatment but also endogenous type I IFN may exert immunoregulatory effects on CD49d, either directly or by indirect effects such as induction of soluble adhesion molecules [24].

The CCR5 and CXCR3 chemokine receptors are expressed on Th1 effector cells and are thought to be involved in the development of inflammatory brain lesions in MS while CCR7 is expressed on naive and central memory T cells. The decrease in CCR5 and CXCR3 expression and the concomitant increase in CCR7 expression observed on CD4+CD26^high^ T cells in MS patients treated with IFN-β in the present study are consistent with reduced activation or a transition of cells within this T cell subset from tissue-homing effector cells to the central memory T cell subset. Indeed, treatment with IFN-β also reduced the expression of CD122 (the IL-2 receptor β-chain), CD134 (OX40), CD137 (4–1BB) and CD212 (the IL-12 receptor β_2_ chain) on CD4+ CD26^high^ T cells.

The observation of an increased percentage of HLA-DR and CD71 positive CD4+ T cells after *in vivo* treatment with IFN-β is a novel observation, which was confirmed *in vitro* for the CD71 induction on CD4+ T cells. IFN-β has previously been reported to induce the expression of activation markers on CD8+ T cells in MS [21], [25]. This is consistent with the results of studies demonstrating that type I IFN can enhance antiviral cytotoxic T cell and NK cell responses, i.e., an immune activating effect of type I IFN [1], [26]. Furthermore, although the *ex vivo* proliferation of CD4+ T cells is suppressed by type I IFNs, *in vivo* CD4+ T cell responses to some viruses are markedly enhanced by type I IFN signalling, and type I IFNs can promote the survival of T cells and enhance the differentiation of memory Th1 cells [27]; [28]–[30].

In our study the percentage of CD4+ HLA-DR+ T cells was an independent predictor of relapse risk both in patients from whom samples were obtained 9–12 hours and in patients from whom samples were obtained 36–48 hours after an injection of IFN-β. In the latter cohort this relationship was highly significant in patients studied within the first five years from onset of MS and during the first two years of treatment but not in the full patient material. This is likely to reflect that a more pristine patient population may be less selected than patients on long-term therapy, and therefore better suited for biomarker studies of the treatment response in MS.

We observed a negative correlation between the percentage of CD4+CD25^high^ T cells expressing CD137 and clinical and MRI disease activity, consistent with the notion that this marker may reflect regulatory T cell activity [31]. We did, indeed, find that *in vitro* treatment with IFN-β increased the percentage of CD4+ T cells that were CD25^high^ and expression of *FOXP3* mRNA. It should, however, be emphasized that the CD4+CD25^high^ T cell subset in MS consists of a mixture of highly activated effector cells and regulatory T cells, and functional studies are needed to substantiate a possible regulatory effect of CD4+CD25^high^ T cells expressing CD137 in MS [13].

We observed a relationship between CD40 expression on dendritic cells and relapse risk in patients from whom blood samples were obtained 9–12 hours after an injection of IFN-β, but this was not confirmed in samples obtained 36–48 hours after an injection of IFN-β. The number of circulating dendritic cells was significantly reduced at the early time point and additional studies using more sophisticated phenotyping of dendritic cell subsets are necessary to establish the relationship between the effects on these cells and disease activity. We could not confirm that the induction of CD40, CD86 and PD-L2 on monocytes is associated with a beneficial response to treatment with IFN-β [32].

Finally the results highlight the importance of consistent timing of blood sampling in relation to IFN-β injections as many changes observed 9–12 hours after an injection of IFN-β were not observed 36–48 hours post injection. The effects of IFN-β on gene expression are mainly transient, and some of these may translate into transient changes in the expression of cell surface molecules [18], [33], [34]. We observed a marked reduction in expression of the chemokine receptor CXCR3 expression 9–12 hours after an injection of IFN-β in all CD4+ T cell subsets studies. This may result from increases in plasma concentrations of the CXCR3 ligand CXCL10 [35], [36]. Indeed, in untreated MS patients we found that the expression of *CXCL10* mRNA correlated with the expression of the type I IFN-induced *MX1* molecule. Furthermore, high *CXCL10* mRNA expression correlated with low CXCR3 expression on CD4+ T cells, suggesting that CXCL10 induced by endogenous type I IFN may regulate physiological T cell expression of CXCR3.

We conclude that the modulation of CD49d and other molecules on CD4+CD26high T cells may be one of the most important effects of IFN-β in MS, and may also be mediated by endogenous type I IFN activity. Conversely, the induction of HLA-DR and CD71 may reflect an unwanted, immune activating effect of IFN-β associated with an increased relapse risk. The induction of CD71 by IFN-β was preventable by combination treatment with methylprednisolone *in vitro*. It is tempting to speculate that this may contribute to the efficacy of combination therapy with methylprednisolone and IFN-β, which significantly reduces the relapse rate in MS compared with IFN-β alone [37], [38]. However, these conclusions are based on studies in small patient cohorts. Larger studies identifying the mechanisms underlying the relationship between T cell activation induced by IFN-β and relapse risk will be important not only for improving our understanding of this immunomodulatory MS therapy, but also for understanding what may be a natural, immunoregulatory role of type I IFN activity in MS.

## Supporting Information

Figure S1 **Principles for flow cytometry analysis.** CD4+ T cells were idenfied according to light scatter and anti-CD4 antibody fluorescence intensity, and were subdivided into a CD25^high^ and a CD26^high^ subset according to anti-CD25 and anti-CD26 fluorescence intensity. Finally, the percentage of CD4+ T cells, CD25^high^ and CD26^high^ CD4+ T cells expressing a panel of antigens was measured against an isotype control antibody (anti-CD49d staining in this example).
(DOC)

Figure S2 **Immune activation in untreated multiple sclerosis.** The percentage of CD4+CD26^high^ T cells expressing CCR5 and the percentage of dendritic cells expressing CD80 was significantly higher in untreated multiple sclerosis without (Gd-) and with (Gd+) gadolinium-enhancing lesions magnetic resonance imaging lesions in the brain than in healthy control subjects. Statistical testing was by the Mann-Whitney U-test.
(DOC)

Table S1 **Surface markers studied by flow cytometry.** This table lists the molecules studied by flow cytometry and their biological functions on T cells and antigen-presenting cells.
(DOC)

Table S2 **Flow cytometry results.** Circulating cell counts, CD4+ T cell subsets, monocytes and dendritic cells in untreated MS patients (n = 39) and patients treated with interferon-β. Blood samples were obtained either 9–12 hours (early, n = 23) or 36–48 hours (late, n = 40) post-injection. Values are medians (inter-quartile range). Statistical testing was by Kruskal-Wallis tests for comparing the three groups groups. Mann-Whitney U-tests were used for post-hoc analysis with Bonferroni-corrected p-values (comparing each treatment group with untreated patients). NS = not significant. p<0.05*, p<0.01**, p<0.001***
(DOC)

## References

[1] Stetson DB, Medzhitov R (2006). Type I interferons in host defense.. Immunity.

[2] Dhib-Jalbut S, Marks S (2010). Interferon-beta mechanisms of action in multiple sclerosis.. Neurology.

[3] Jacobs LD, Cookfair DL, Rudick RA, Herndon RM, Richert JR (1996). Intramuscular interferon beta-1a for disease progression in relapsing multiple sclerosis. The Multiple Sclerosis Collaborative Research Group (MSCRG).. Ann Neurol.

[4] PRISMS (Prevention of Relapses and Disability by Interferon beta-1a Subcutaneously in Multiple Sclerosis) Study Group (1998). Randomised double-blind placebo-controlled study of interferon beta-1a in relapsing/remitting multiple sclerosis.. Lancet.

[5] The IFNB Multiple Sclerosis Study Group (1993). Interferon beta-1b is effective in relapsing-remitting multiple sclerosis. I. Clinical results of a multicenter, randomized, double-blind, placebo-controlled trial.. Neurology.

[6] van Baarsen LG, van der Pouw Kraan TC, Kragt JJ, Baggen JM, Rustenburg F (2006). A subtype of multiple sclerosis defined by an activated immune defense program.. Genes Immun.

[7] Yamaguchi KD, Ruderman DL, Croze E, Wagner TC, Velichko S (2008). IFN-beta-regulated genes show abnormal expression in therapy-naive relapsing-remitting MS mononuclear cells: gene expression analysis employing all reported protein-protein interactions.. J Neuroimmunol.

[8] Hesse D, Krakauer M, Lund H, Søndergaard HB, Langkilde A (2010). Breakthrough disease during interferon-beta therapy in MS: No signs of impaired biologic response.. Neurology.

[9] Hesse D, Krakauer M, Lund H, Søndergaard HB, Limborg SJ (2011). Disease protection and interleukin-10 induction by endogenous interferon-beta in multiple sclerosis?. Eur J Neurol.

[10] van der Voort LF, Vennegoor A, Visser A, Knol DL, Uitdehaag BM (2010). Spontaneous MxA mRNA level predicts relapses in patients with recently diagnosed MS.. Neurology.

[11] Comabella M, Lunemann JD, Rio J, Sánchez A, López C (2009). A type I interferon signature in monocytes is associated with poor response to interferon-beta in multiple sclerosis.. Brain.

[12] Baecher-Allan C, Brown JA, Freeman GJ, Hafler DA (2001). CD4+CD25high regulatory cells in human peripheral blood.. J Immunol.

[13] Michel L, Berthelot L, Pettre S, Wiertlewski S, Lefrère F (2008). Patients with relapsing-remitting multiple sclerosis have normal Treg function when cells expressing IL-7 receptor alpha-chain are excluded from the analysis.. J Clin Invest.

[14] Jensen J, Langkilde AR, Fenst C, Nicolaisen MS, Roed HG (2004). CD4 T cell activation and disease activity at onset of multiple sclerosis.. J Neuroimmunol.

[15] Krakauer M, Sorensen PS, Sellebjerg F (2006). CD4(+) memory T cells with high CD26 surface expression are enriched for Th1 markers and correlate with clinical severity of multiple sclerosis.. J Neuroimmunol.

[16] Hafler DA, Fox DA, Manning ME, Schlossman SF, Reinherz EL, Weiner HL (1985). In vivo activated T lymphocytes in the peripheral blood and cerebrospinal fluid of patients with multiple sclerosis.. N Engl J Med.

[17] Khoury SJ, Guttmann CR, Orav EJ, Kikinis R, Jolesz FA, Weiner HL (2000). Changes in activated T cells in the blood correlate with disease activity in multiple sclerosis.. Arch Neurol.

[18] Sellebjerg F, Krakauer M, Hesse D, Ryder LP, Alsing I (2009). Identification of new sensitive biomarkers for the in vivo response to interferon-beta treatment in multiple sclerosis using DNA-array evaluation.. Eur J Neurol.

[19] Weinstock-Guttman B, Bhasi K, Badgett D, Tamaño-Blanco M, Minhas M (2008). Genomic effects of once-weekly, intramuscular interferon-beta1a treatment after the first dose and on chronic dosing: Relationships to 5-year clinical outcomes in multiple sclerosis patients.. J Neuroimmunol.

[20] Jensen J, Krakauer M, Sellebjerg F (2005). Cytokines and adhesion molecules in multiple sclerosis patients treated with interferon-beta1b.. Cytokine.

[21] Jensen J, Langkilde AR, Frederiksen JL, Sellebjerg F (2006). CD8+ T cell activation correlates with disease activity in clinically isolated syndromes and is regulated by interferon-beta treatment.. J Neuroimmunol.

[22] Sellebjerg F, Ross C, Koch-Henriksen N, Sørensen PS, Frederiksen JL (2005). CD26+ CD4+ T cell counts and attack risk in interferon-treated multiple sclerosis.. Mult Scler.

[23] Barrau MA, Montalban X, Saez-Torres I, Brieva L, Barbera N (2000). CD4(+)CD45RO(+)CD49d(high) cells are involved in the pathogenesis of relapsing-remitting multiple sclerosis.. J Neuroimmunol.

[24] Calabresi PA, Pelfrey CM, Tranquill LR, Maloni H, McFarland HF (1997). VLA-4 expression on peripheral blood lymphocytes is downregulated after treatment of multiple sclerosis with interferon beta.. Neurology.

[25] Ossege LM, Sindern E, Patzold T, Malin JP (2001). Immunomodulatory effects of interferon-beta-1b in patients with multiple sclerosis.. Int Immunopharmacol.

[26] Tough DF, Borrow P, Sprent J (1996). Induction of bystander T cell proliferation by viruses and type I interferon in vivo.. Science.

[27] Havenar-Daughton C, Kolumam GA, Murali-Krishna K (2006). Cutting Edge: The direct action of type I IFN on CD4 T cells is critical for sustaining clonal expansion in response to a viral but not a bacterial infection.. J Immunol.

[28] Krug A, Veeraswamy R, Pekosz A, Kanagawa O, Unanue ER (2003). Interferon-producing cells fail to induce proliferation of naive T cells but can promote expansion and T helper 1 differentiation of antigen-experienced unpolarized T cells.. J Exp Med.

[29] Lombardi G, Dunne PJ, Scheel-Toellner D, Sanyal T, Pilling D (2000). Type 1 IFN maintains the survival of anergic CD4+ T cells.. J Immunol.

[30] Marrack P, Kappler J, Mitchell T (1999). Type I interferons keep activated T cells alive.. J Exp Med.

[31] Weinberg AD, Montler R (2005). Modulation of TNF receptor family members to inhibit autoimmune disease.. Curr Drug Targets Inflamm Allergy.

[32] Wiesemann E, Deb M, Trebst C, Hemmer B, Stangel M, Windhagen A (2008). Effects of interferon-beta on co-signaling molecules: upregulation of CD40, CD86 and PD-L2 on monocytes in relation to clinical response to interferon-beta treatment in patients with multiple sclerosis.. Mult Scler.

[33] Sellebjerg F, Datta P, Larsen J, Rieneck K, Alsing I (2008). Gene expression analysis of interferon-beta treatment in multiple sclerosis.. Mult Scler.

[34] Weinstock-Guttman B, Badgett D, Patrick K, Hartrich L, Santos R (2003). Genomic effects of IFN-beta in multiple sclerosis patients.. J Immunol.

[35] Buttmann M, Merzyn C, Hofstetter HH, Rieckmann P (2007). TRAIL, CXCL10 and CCL2 plasma levels during long-term Interferon-beta treatment of patients with multiple sclerosis correlate with flu-like adverse effects but do not predict therapeutic response.. J Neuroimmunol.

[36] Krakauer M, Sorensen PS, Khademi M, Olsson T, Sellebjerg F (2006). Dynamic T-lymphocyte chemokine receptor expression induced by interferon-beta therapy in multiple sclerosis.. Scand J Immunol.

[37] Ravnborg M, Sorensen PS, Andersson M, Celius EG, Jongen PJ (2010). Methylprednisolone in combination with interferon beta-1a for relapsing-remitting multiple sclerosis (MECOMBIN study): a multicentre, double-blind, randomised, placebo-controlled, parallel-group trial.. Lancet Neurol.

[38] Sorensen PS, Mellgren SI, Svenningsson A, Elovaara I, Frederiksen JL (2009). NORdic trial of oral Methylprednisolone as add-on therapy to Interferon beta-1a for treatment of relapsing-remitting Multiple Sclerosis (NORMIMS study): a randomised, placebo-controlled trial.. Lancet Neurol.

