# Supplementary figures and images for: Endogenous and Recombinant Type I Interferons and Disease Activity in Multiple Sclerosis
Source: PLoS One. 2012 Jun 6;7(6):e35927. doi: 10.1371/journal.pone.0035927 (PMC3368920; doi:10.1371/journal.pone.0035927)

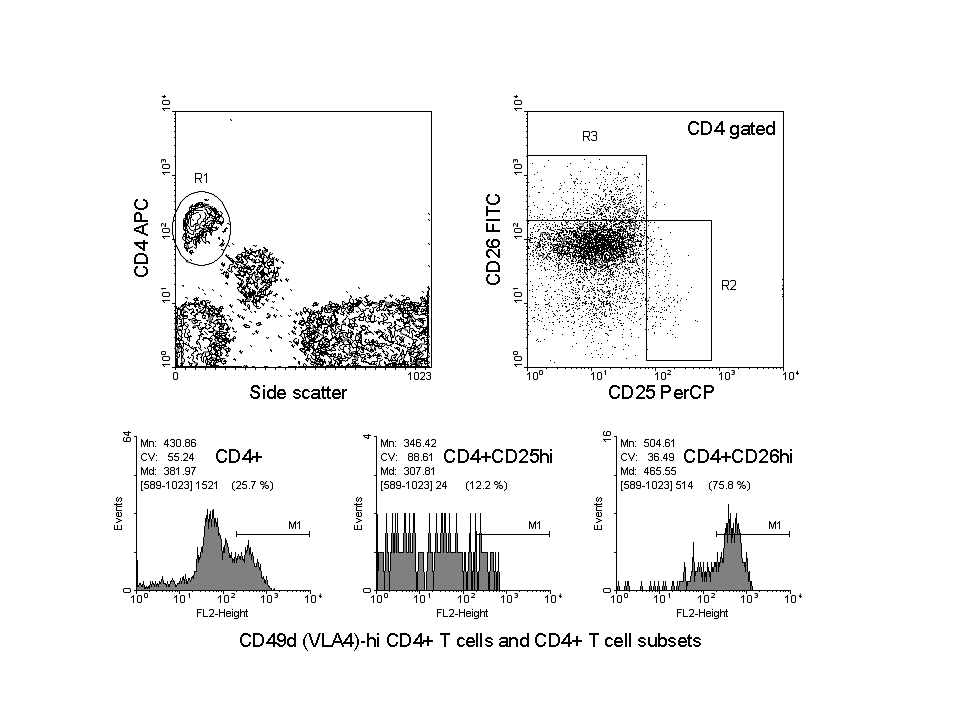

Supplement: Figure S1 — Principles for flow cytometry analysis. CD4+ T cells were idenfied according to light scatter and anti-CD4 antibody fluorescence intensity, and were subdivided into a CD25high and a CD26high subset according to anti-CD25 and anti-CD26 fluorescence intensity. Finally, the percentage of CD4+ T cells, CD25high and CD26high CD4+ T cells expressing a panel of antigens was measured against an isotype control antibody (anti-CD49d staining in this example). (DOC) [file pone.0035927.s001.doc]

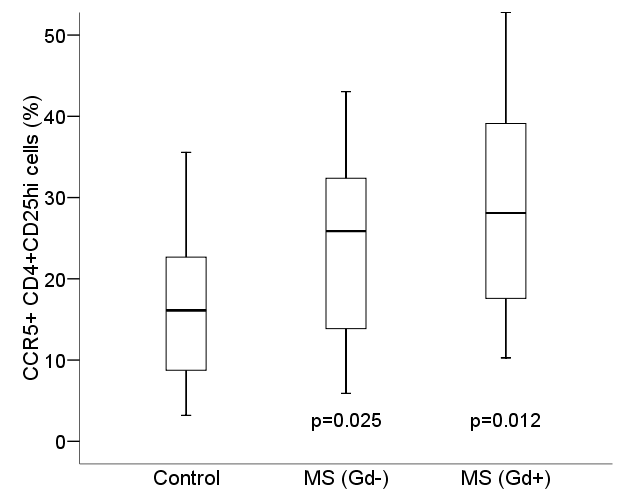


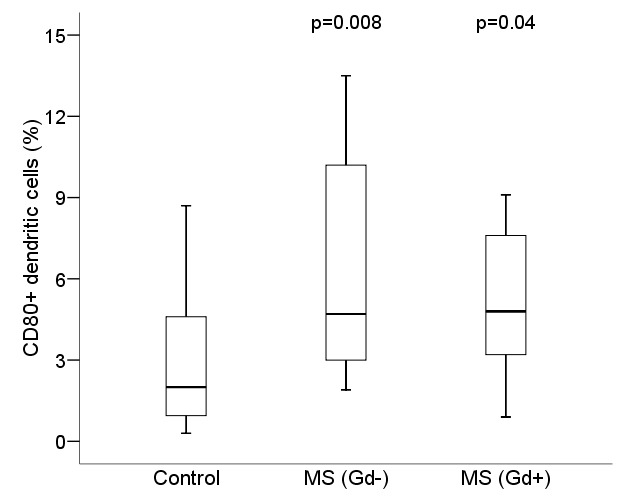

Supplement: Figure S2 — Immune activation in untreated multiple sclerosis. The percentage of CD4+CD26high T cells expressing CCR5 and the percentage of dendritic cells expressing CD80 was significantly higher in untreated multiple sclerosis without (Gd-) and with (Gd+) gadolinium-enhancing lesions magnetic resonance imaging lesions in the brain than in healthy control subjects. Statistical testing was by the Mann-Whitney U-test. (DOC) [file pone.0035927.s002.doc]
