# Supplementary material for: Endogenous and Recombinant Type I Interferons and Disease Activity in Multiple Sclerosis
Source: PLoS One. 2012 Jun 6;7(6):e35927. doi: 10.1371/journal.pone.0035927 (PMC3368920; doi:10.1371/journal.pone.0035927)
Supplement: Table S1 — Surface markers studied by flow cytometry. This table lists the molecules studied by flow cytometry and their biological functions on T cells and antigen-presenting cells. (DOC) [file pone.0035927.s003.doc]

Antigen-presenting cells

CD40 Receptor for activating signal from CD154 (CD40-ligand)

CD80 (B7-1) Costimulatory molecule

CD86 (B7-2) Costimulatory molecule

CD195 (CCR5) Receptor for chemokines CCL3, CCL4, CCL5 and CCL8

T cells

Molecule name(s)

CD49d α chain of α4β1 (VLA4) and α4β7 integrin (adhesion molecules)

CD71 Transferrin receptor expressed on activated lymphocytes

CD95 (Fas) Receptor for Fas-ligand – induction of apoptosis

CD122 β-chain of the interleukin-2 receptor complex

CD134 (OX-40) Costimulatory molecule

CD137 (4-1BB) Costimulatory molecule

CD162 (PSGL-1) Platelet selectin glycolipid ligand-1 (adhesion molecule)

CD178 Fas-ligand – induction of apoptosis

CD183 (CXCR3) Receptor for chemokines CXCL9, CXCL10, CXCL11

CD194 (CCR4) Receptor for chemokines CCL17 and CCL22

CD195 (CCR5) Receptor for chemokines CCL3, CCL4 and CCL5 and CCL8

CD197 (CCR7) Receptor for chemokines CCL19 and CCL21

CD212 β_2_ subunit of the interleukin-12 receptor (expressed on Th1 cells)

GITR Glucocorticoid-inducible TNFR-related gene (*TNFRSF18*)

HLA-DR Human leukocyte antigen DR molecule
