# Supplementary material for: Endogenous and Recombinant Type I Interferons and Disease Activity in Multiple Sclerosis
Source: PLoS One. 2012 Jun 6;7(6):e35927. doi: 10.1371/journal.pone.0035927 (PMC3368920; doi:10.1371/journal.pone.0035927)
Supplement: Table S2 — Flow cytometry results. Circulating cell counts, CD4+ T cell subsets, monocytes and dendritic cells in untreated MS patients (n = 39) and patients treated with interferon-β. Blood samples were obtained either 9–12 hours (early, n = 23) or 36–48 hours (late, n = 40) post-injection. Values are medians (inter-quartile range). Statistical testing was by Kruskal-Wallis tests for comparing the three groups groups. Mann-Whitney U-tests were used for post-hoc analysis with Bonferroni-corrected p-values (comparing each treatment group with untreated patients). NS = not significant. p<0.05*, p<0.01**, p<0.001*** (DOC) [file pone.0035927.s004.doc]

Untreated IFN-β, early IFN-β, late

# Circulating cell counts

Lymphocytes x 10^9^/l (p<0.001) 1.80 (0.60) 0.80 (0.50)*** 1.75 (0.50) ^NS^

Monocytes x 10^9^/l (p=0.001) 0.40 (0.13) 0.58 (0.13)*** 0.47 (0.18) ^NS^

Dencritic cells x 10^6^/l (p<0.001) 11.2 (7.3) 5.4 (3.8)*** 9.6 (4.9) ^NS^

Plasmacytoid dendritic 4.7 (4.2) 3.1 (3.3)* 5.2 (4.2) ^NS^

cells x 10^6^/l (p=0.012)

Myeloid dendritic 4.3 (2.6) 1.7 (1.4)*** 3.2 (1.7) ^NS^

cells x 10^6^/l (p<0.001)

CD3+ T cells x 10^9^/l (p<0.001) 1.12 (0.39) 0.52 (0.29)*** 1.22 (0.42) ^NS^

CD4+ T cells x 10^9^/l (p<0.001) 0.85 (0.37) 0.37 (0.17)*** 0.83 (0.32) ^NS^

# T cell activation and differentiation

CD25^high^ CD4+ T cells (%, p=0.005) 2.4 (0.8) 3.9 (1.9)** 2.7 (1.1) ^NS^

CD26^high^ CD4+ T cells (%, NS) 12.9 (6.3) 11.4 (4.1) 10.9 (6.2)

CD71 (% of subset)

CD4 (p=0.001) 5.6 (3.2) 7.9 (3.9)** 8.3 (3.9)**

CD4+ CD25^hi^ (p=0.003) 27 (14) 33 (12)** 35 (14)*

CD4+ CD26^hi^ (NS) 11 (5.1) 11 (7.6) 13 (6.2)

CD95 (Fas; % of subset)

CD4 (NS) 30 (10) 32 (14) 24 (13)

CD4+ CD25^hi^ (NS) 82 (12) 82 (11) 75 (14)

CD4+ CD26^hi^ (NS) 38 (14) 42 (17) 34 (10)

CD122 (IL-2Rβ; % of subset)

CD4 (NS) 2.7 (1.6) 2.5 (1.6) 2.4 (0.8)

CD4+ CD25^hi^ (NS) 3.7 (3.5) 2.6 (2.3) 2.9 (2.8)

CD4+ CD26^hi^ (0.039) 9.6 (5.8) 8.1 (5.2) ^NS^ 7.1 (3.6)*

CD134 (OX-40; % of subset)

CD4 (NS) 4.7 (2.5) 4.1 (2.1) 3.6 (2.2)

CD4+ CD25^hi^ (NS) 2.3 (3.3) 2.7 (1.6) 2.8 (1.3)

CD4+ CD26^hi^ (p=0.041) 17 (8.8) 14 (5.6) ^NS^ 12 (7.8)*

CD137 (4-1BB; % of subset)

CD4 (NS) 4.6 (2.6) 3.7 (2.3) 4.0 (1.6)

CD4+ CD25^hi^ (p<0.001) 21 (10) 12 (6.4)*** 17 (6.5) ^NS^

CD4+ CD26^hi^ (p=0.049) 12 (6.9) 9.8 (8.0) ^NS^ 8.6 (4.3)*

CD178 (Fas-ligand; % of subset)

CD4 (NS) 3.1 (2.0) 2.9 (1.2) 3.1 (1.4)

CD4+ CD25^hi^ (NS) 1.4 (1.8) 1.5 (1.7) 1.9 (1,8)

CD4+ CD26^hi^ (NS) 11 (5.7) 9.5 (6.6) 9.3 (5.4)

CD212 (IL-12R-β2; % of subset)

CD4 (NS) 2.5 (1.3) 2.1 (1.3) 2.3 (0.8)

CD4+ CD25^hi^ (NS) 1.2 (1.2) 1.2 (1.4) 0.9 (0.9)

CD4+ CD26^hi^ (p=0.036) 10 (4.7) 8.0 (5.6) 7.7 (3.8)*

GITR (% of subset)

CD4 (NS) 5.1 (2.3) 4.3 (3.5) 4.1 (2.5)

CD4+ CD25^hi^ (p=0.017) 4.2 (3.1) 4.4 (5.0) ^NS^ 2.6 (3.0)*

CD4+ CD26^hi^ (NS) 14 (7.3) 11 (7.8) 11 (5.6)

HLA-DR (% of subset)

CD4 (p=0.001) 8.9 (2.9) 11.3 (6.5)** 10.9 (3.8)**

CD4+ CD25^hi^ (NS) 52 (9.5) 51 (13) 52 (12)

CD4+ CD26^hi^ (NS) 13 (6.3) 12 (7.2) 12 (4.5)

# Adhesion molecules and chemokine receptors

CD49d^hi^ (VLA4^hi^; % of subset)

CD4 (NS) 33 (13) 30 (12) 28 (13)

CD4+ CD25^hi^ (NS) 15 (9.7) 14 (7.9) 14 (7.2)

CD4+ CD26^hi^ (p=0.003) 72 (14) 65 (9.8)* 64 (15)**

CD62L (L-selectin; % of subset)

CD4 (NS) 83 (7.6) 84 (6.2) 86 (7.6)

CD4+ CD25^hi^ (NS) 89 (7.7) 92 (7.0) 90 (8.0)

CD4+ CD26^hi^ (p=0.030) 55 (12) 64 (13)* 58 (13) ^NS^

CD162 (PSGL1, % of subset)

CD4 (NS) 42 (14) 45 (17) 41 (13)

CD4+ CD25^hi^ (NS) 85 (9.0) 85 (9.1) 86 (8.3)

CD4+ CD26^hi^ (NS) 75 (15) 72 (17) 68 (9.2)

CD183 (CXCR3, % of subset)

CD4 (p<0.001) 31 (13) 16 (12)*** 27 (9.9) ^NS^

CD4+ CD25^hi^ (p<0.001) 31 (11) 15 (11)*** 29 (11) ^NS^

CD4+ CD26^hi^ (p<0.001) 68 (12) 35 (23)*** 60 (12)***

CD194 (CCR4; % of subset)

CD4 (NS) 13 (6.2) 14 (8.0) 14 (6.6)

CD4+ CD25^hi^ (NS) 50 (17) 41 (22) 54 (17)

CD4+ CD26^hi^ (NS) 17 (8.5) 16 (6.1) 17 (6.3)

CD195 (CCR5; % of subset)

CD4 (p=0.013) 8.5 (5.0) 8.3 (3.9) ^NS^ 6.2 (2.1)*

CD4+ CD25^hi^ (p=0.001) 28 (22) 20 (7.9) ^NS^ 17 (11.6)*

CD4+ CD26^hi^ (p=0.006) 25 (17) 20 (9.4) ^NS^ 19 (8.4)**

CD197 (CCR7; % of subset)

CD4 (NS) 70 (13) 63 (26) 75 (14)

CD4+ CD25^hi^ (NS) 30 (11) 35 (15) 31 (15)

CD4+ CD26^hi^ (p<0.001) 93 (7.9) 88 (23) ^NS^ 96 (5.2)**

# Monocyte activation

CCR5+ (%, p=0.017) 3.2 (2.4) 2.2 (2.2)* 3.6 (2.0)

CD40+ (%, NS) 6.7 (7.1) 9.8 (10.2) 6.7 (6.2)

CD80+ (%, NS) 1.5 (1.0) 2.0 (0.5) 1.8 (0.9)

CD86 (MFI, p<0.001) 2804 (827) 3707 (1617)*** 3859 (1535)***

# Dendritic cell activation

CCR5+ (%, p<0.001) 39 (33) 11 (17)*** 27 (26)**

CD40+ (%, NS) 10 (7.5) 7.3 (5.2) 9.8 (7.1)

CD80+ (%, p=0.039) 4.5 (5.3) 7.2 (9.4) ^NS^ 7.1 (5.7)*

CD86+ (%, p=0.005) 78 (13) 66 (18)** 77 (12) ^NS^
